# Cat-PIPpred: Pro-Inflammatory Peptide Predictor Integrating CatBoost and Cross-Modal Feature Fusion

**DOI:** 10.3390/ijms262110484

**Published:** 2025-10-28

**Authors:** Jia Zheng, Xianmian Qin, Yue Yao

**Affiliations:** School of Science, Dalian Maritime University, Dalian 116026, China

**Keywords:** pro-inflammatory peptides, feature integration, feature refinement, ensemble learning

## Abstract

Pro-inflammatory peptides (PIPs) play a pivotal role in the initiation, progression, and sustenance of inflammation. A more in-depth analysis of PIPs requires precise identification, for which computational methodologies have proven to be remarkably cost-effective and accurate. In this study, we introduce Cat-PIPpred, a sophisticated predictor for PIPs that combines CatBoost with cross-modal feature integration. Through a comprehensive evaluation involving cross-validation and independent testing, an optimized model is developed by employing various feature extraction techniques, refinement protocols, and classifier architectures. The integration of ESM-2 structural embeddings with Dipeptide Deviation from Expected Mean (DDE) evolutionary features allows for an extensive representation of sequences. Feature refinement effectively decreases memory consumption while enhancing operational efficiency. The final Cat-PIPpred surpasses existing predictors targeting PIPs, as well as general peptide classifiers. These findings affirm the efficacy of integrating multiple feature sets with advanced ensemble learning algorithms. The proposed framework not only ensures reliable PIP predictions but also offers valuable insights into the functional predictions of specialized peptides.

## 1. Introduction

Inflammation is a complex immune response to infection, injury, and other harmful stimuli, which not only initiates certain biological processes but also facilitates interactions among various cells and molecules [1]. Acute inflammation serves as a vital physiological defense mechanism. Persistent inflammation accelerates cellular damage, disrupts tissue homeostasis, and contributes to major disease progression, including cancer, cardiovascular diseases, autoimmune disorders, neurodegenerative conditions and so on [2]. Extensive investigation has been prompted into alternative anti-inflammatory approaches with enhanced selectivity. An in-depth investigation and enhanced understanding of inflammation will facilitate the development and refinement of anti-inflammatory strategies.

Pro-inflammatory peptides (PIPs) are crucial in the initiation, progression, and maintenance of inflammation. PIPs can induce cells to secrete multiple pro-inflammatory cytokines, such as TNF-α, IL-1, and IL-6 [3,4]. These cytokines form a positive feedback loop at inflammatory sites, further exacerbating the inflammatory response. Therefore, deeper research into the biological functions and mechanisms of PIPs is of significant importance for the development of alternative anti-inflammatory therapeutic strategies [5], and the accurate prediction and identification of PIPs represents a critical research priority.

Algorithm-driven identification and prediction approaches could be cost-effective, time-efficient and highly accurate. Several computational tools have been developed for predicting PIPs, primarily utilizing sequence-based features. Among existing tools, ProInflam implements SVM-based algorithms incorporating hybrid features that combine dipeptide composition (DPC) with motif information derived from the Betts–Russell algorithm [6]. PIP-EL employs an ensemble machine learning approach comprising 50 independent random forest (RF) models [7]. Its feature set incorporates amino acid composition (AAC), DPC, composition–transition–distribution (CTD), physicochemical properties (PCP), and amino acid index (AA index). ProIn-Fuse utilizes eight sequence encoding schemes to generate probabilistic scores through RF models, including k-mer composition from profile (Kmer-pr), profile based composition of the amino acid (PKA), k-mer composition of the amino acid (Kmer-ac), k-space amino acid pairs (KSAP), binary encoding (BE), amino acid index properties (AIP), N-terminal 5 and C-terminal 5 dipeptides composition (C5N5-DC), and structural features (SF) [8]. MultiFeatVotPIP integrates AAC, DPC, AA index, dipeptide deviation from expected mean (DDE), and grouped dipeptide composition (GDPC) features, employing a soft voting ensemble framework with five core learners (AdaBoost, XGBoost, RF, GBDT, and LightGBM) [9]. Additional feature encoding schemes have been evaluated, including Composition of K-Spaced Amino Acid Group Pairs (CKSAAGP), Composition-Transition-Distribution of Codons (CTDC), Generalized Topological Polar Coefficient (GTPC), Composition-Transition-Distribution of Tripeptides (CTDT), and Pseudo Amino Acid Composition (PAAC). Notably, AA index and DDE features demonstrate superior discriminative capability for PIP identification. Detailed analytical studies have revealed distinct compositional and positional preferences of amino acid residues between PIPs and non-PIPs, highlighting the importance of sequence information for PIP identification. Additionally, various other machine learning algorithms have been assessed, including Extremely Randomized Trees (ERT), naïve Bayes (NB), K Nearest Neighbors (KNN), Logistic Regression (LR), Stochastic Gradient Descent (SGD), Linear Discriminant Analysis (LDA), Convolutional Neural Network (CNN), Long Short-Term Memory (LSTM), BiLSTM, Transformer, Bert_CNN, and LSTM Attention. They were ultimately excluded from final model consideration due to inferior predictive performance.

These approaches have witnessed the development of PIP prediction, showcasing the potential of machine learning-driven innovations in bioinformatics. Nevertheless, the predictive performance of existing models remains suboptimal, highlighting the necessity for further refinement. The ESM-2 model [10] demonstrates strong capability in extracting global contextual structure features from peptide sequences, and has been empirically validated as an effective tool for predicting peptide functional activities [11]. The integration of advanced learning method and enhanced feature representations may help improve prediction performance.

**Figure 1:**
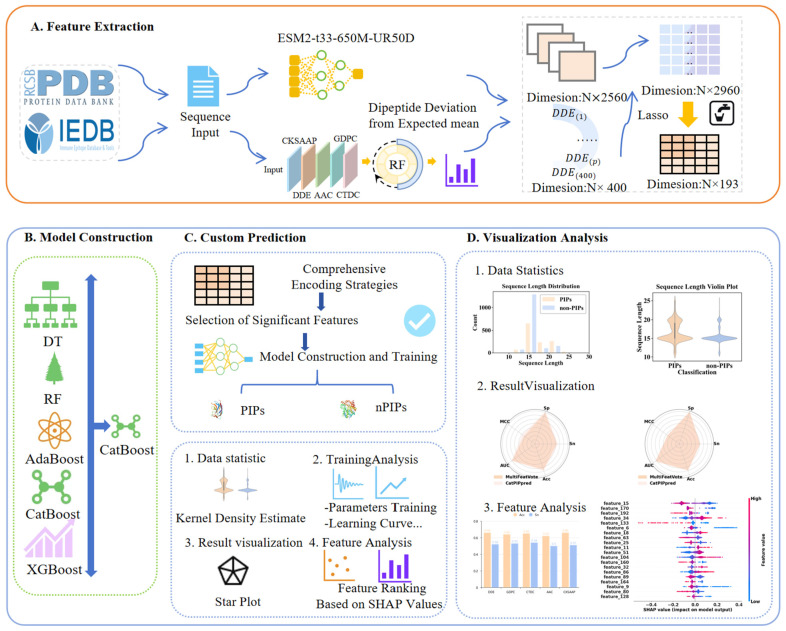
Overall workflow of Cat-PIPpred.

In this work, we propose Cat-PIPpred, an advanced computational framework for improved prediction of PIPs. Our approach involves the following: (1) Dual-encoding PIP and non-PIP sequences using protein language model (ESM-2) and evolutionary feature representations (DDE); ESM-2 is employed to capture high-level structural features of peptides, while DDE encoding is used to represent the implicit evolutionary relationships. (2) Integration and refinement of hybrid feature vectors for final classification. Features are selected and refined before being processed by a CatBoost classifier for PIP identification. Different feature refinement strategies and classification algorithms are evaluated systematically so as to optimize model performance. The final model Cat-PIPpred outperforms existing PIP-targeted predictors and state-of-the-art peptide functional classifiers by evaluating on cross-validation and independent tests. These results highlight the effectiveness of our hybrid feature representation scheme and the superiority of CatBoost architecture for PIP recognition tasks. The overall workflow of this study is illustrated in Figure 1.

## 2. Results

During model development, multiple feature extraction methods, feature refinement strategies, and classifier algorithms are systematically evaluated through 5-fold cross-validation and independent tests to optimize predictive performance.

### 2.1. Feature Evaluation

**Figure 2:**
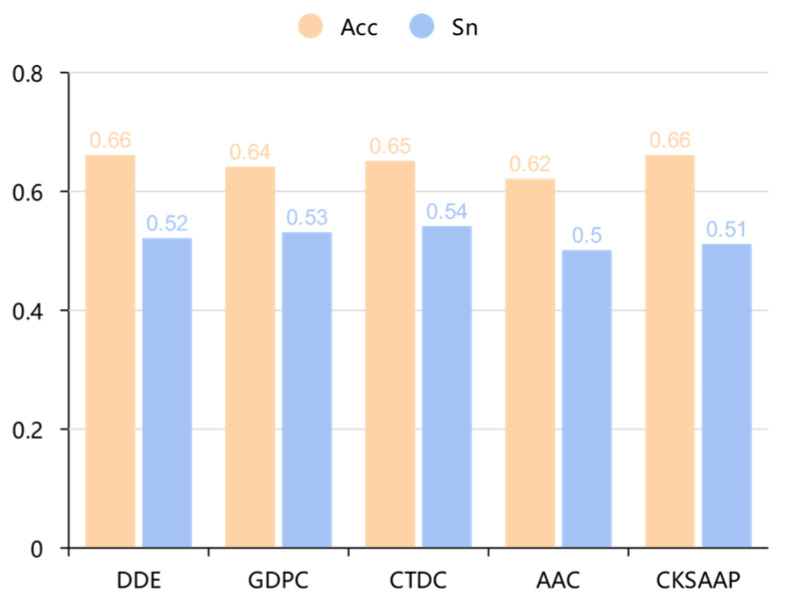
The performances of individual features using RF as a classifier.

The compositional feature representation is chosen out of five sequence-based encoding, including DDE, AAC, GDPC, CTDC, and CKSAAP. Their predictive contributions were evaluated on the training datasets via a RF basic classifier, yielding a feature importance ranking according to their Acc values. As shown in Figure 2, DDE and CKSAAP features lead to the highest Acc value of 0.66, and the Sn value of DDE is higher than that of CKSAAP. Consequently, DDE feature is chosen to represent the compositional properties of peptide sequences.

### 2.2. Feature Refinement and Model Optimization

Both independent tests and 5-fold cross-validation tests are conducted to determine the best combination of feature refinement strategy and classifier. In this study, the hybrid feature of ESM-2 and DDE is 2960 (2560 + 400) dimensions. To optimize model performance, feature selection was implemented using Lasso regression for dimensionality reduction. For the LASSO regularization, the penalty strength was optimized using 10-fold cross-validation, with the random seed fixed at 42.

Various models were constructed based on different learning algorithms. On the five-fold cross-validation experiments, combinations of different feature refinement strategies and machine learning algorithms exhibited significant performance variations, as listed in Table 1.

Overall, models based on Two-stage (526 dimensions) and Dual-channel (210 dimensions) feature refinement demonstrated superior comprehensive performance. Notably, the combination of Dual-channel features with the CatBoost algorithm achieved the highest levels in Acc of 0.700 and MCC of 0.381, while the combination of Two-stage features with XGBoost also showed strong performance in Acc of 0.696 and AUC of 0.741.

The assessment on the independent test dataset is listed in Table 2, and the combination of Global feature refinement with CatBoost exhibited the best comprehensive performance on the independent test (Acc = 0.684, MCC = 0.381), with its Sp of 0.813 being significantly higher than other combinations, suggesting strong robustness in identifying negative samples. It is noteworthy that the combination of Two-stage features with XGBoost maintained relatively balanced performance on the independent test (Acc = 0.655, Sn = 0.556, MCC = 0.316).

By integrating performance insights from both independent testing and five-fold cross-validation, we developed Cat-PIPpred, an optimized predictive framework that synergistically combines global feature refinement with the CatBoost algorithm. The CatBoost model was configured with a learning rate of 0.1, a tree depth of 4, and 100 iterations.

Considering that CKSAAP encoding performed second best in Section 2.1 (with a minimal difference of 0.01 in Sn), we systematically investigated whether this subtle difference would be amplified in downstream modeling through a comprehensive comparison between DDE and CKSAAP encoding schemes. We replaced DDE with CKSAAP and conducted 5-fold cross-validation and independent tests, while maintaining all other settings, including the same ESM-2 features and model pipeline. Table 3 shows the 5-fold cross-validation results, and Table 4 presents the results of independent tests. Among all evaluated methods, XGBoost with Global feature refinement demonstrated superior overall performance, achieving the highest scores in 4 out of 5 metrics in the 5-fold cross-validation tests. Moreover, this combination attained the highest Acc (0.678), AUC (0.730), and MCC (0.366) on the independent test set. Based on these results, we constructed an alternative model with CKSAAP encoding, termed CKSAAP_XG-PIPpred, which utilizes XGBoost with the Global feature refinement strategy, in contrast to the DDE-based Cat-PIPpred model.

Table 5 compares the performance of Cat-PIPpred and CKSAAP_XG-PIPpred. In 5-fold cross-validation, CKSAAP_XG-PIPpred outperformed Cat-PIPpred in 4 out of 5 metrics, with particularly notable advantages in Sn (0.606 vs. 0.520, +0.086), AUC (0.778 vs. 0.738, +0.040) and MCC (0.397 vs. 0.361, +0.036). However, in the independent test, Cat-PIPpred demonstrated better performance in Acc (0.684 vs. 0.678, +0.006), Sp (0.813 vs. 0.789, +0.024), and MCC (0.381 vs. 0.366, +0.015). Notably, Cat-PIPpred exhibited more stable performance across different evaluation scenarios. Cat-PIPpred maintained consistent performance with improved Sn (0.556 vs. 0.520) and MCC (0.381 vs. 0.361) on the independent dataset, while CKSAAP_XG-PIPpred showed significant performance degradation from cross-validation to independent testing. These results indicate that Cat-PIPpred possesses stronger generalization capability. Therefore, based on this comprehensive comparison, we conclude that Cat-PIPpred is the optimal model for PIP recognition.

In parallel, we explored deep learning architectures as alternative classifiers for PIP identification, which were also trained and tested using the global refinement strategy. Three distinct convolutional neural network (CNN) architectures were implemented, alongside an Att_BiLSTM network—a hybrid architecture incorporating an attention mechanism followed by a bidirectional LSTM layer with 32 hidden units. Hyper parameters and comparative five-fold cross-validation metrics are detailed in Table 6. Although CNNs achieved performance comparable to traditional machine learning classifiers, none surpassed Cat-PIPpred. The Att_BiLSTM attained peak Sn but demonstrated suboptimal results across other metrics. Crucially, Cat-PIPpred exhibited superior overall efficacy with improvements of 1.0% Acc, 1.4% Sp, and 3.5% AUC versus alternatives. These results establish CatBoost as the optimal approach for PIP identification, while CNNs remain viable context-dependent alternatives.

### 2.3. Comparison with Other Methods

To evaluate the effectiveness of Cat-PIPpred, we conducted comparative analyses using both 5-fold cross-validation and independent test datasets in relation to three established predictors: MultifeatVotPIP, Deep_AMPpred [11], and AMPpred_MFA [12]. In our work, we employed exactly the same training dataset and independent test dataset as MultiFeatVotPIP, and we tested Deep_AMPpred and AMPpred_MFA on these datasets. All competing methods were evaluated on the identical datasets, thus ensuring a fair comparison of the results. The results are outlined in Table 7.

**Figure 3:**
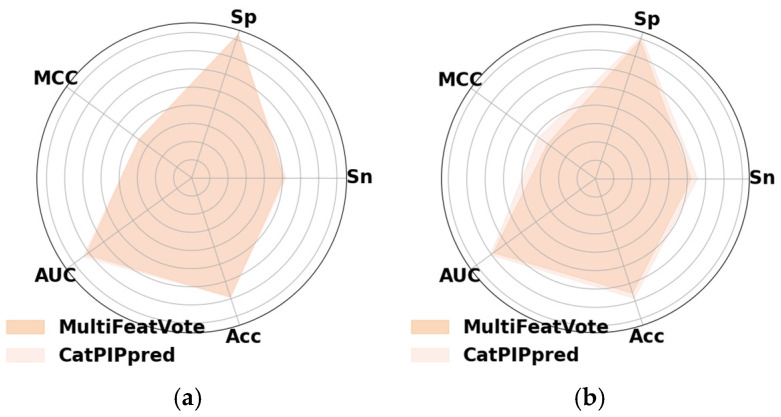
Performance comparison of Cat-PIPpred and MultiFeatVotPIP: (**a**) 5-fold cross-validation; (**b**) independent test.

In the 5-fold cross-validation evaluation, Cat-PIPpred outperformed in three key metrics, achieving an AUC of 0.738, an MCC of 0.361, and a sensitivity of 0.520, while showing comparable accuracy to MultifeatVotPIP with a score of 0.691. Although its specificity was recorded at 0.821, slightly lower than MultifeatVotPIP by 0.9%, it exhibited a 1.2% improvement in sensitivity. In the independent test set, Cat-PIPpred surpassed all other methods in four main metrics: accuracy at 0.684, reflecting a 2.9% enhancement; sensitivity at 0.556, representing a 3.5% increase; AUC at 0.705, showing a 1.9% rise; and MCC at 0.381, indicating a 5.9% improvement. Its specificity of 0.813 placed it second, only behind AMPpred_MFA. Notably, Cat-PIPpred outperformed MultiFeatVotPIP in every metric evaluated in the independent test, as shown in Figure 3.

To evaluate the statistical significance of the performance improvement, we compared Cat-PIPpred against the MultifeatVotPIP baseline using a paired-sample *t*-test on the independent test set. The Shapiro–Wilk test confirmed the data met the normality assumption (*p* = 0.292). The mean performance values are 0.595 ± 0.180 for MultifeatVotPIP and 0.628 ± 0.165 for Cat-PIPpred, with the mean paired difference of −0.033 ± 0.015. The *t*-test results indicate that Cat-PIPpred’s superior performance is statistically significant as t(4) = −4.686 and *p* = 0.009. The magnitude of this difference is substantial, as indicated by a Cohen’s d value of 2.096, which denotes a very large effect size.

## 3. Discussion

**Figure 4:**
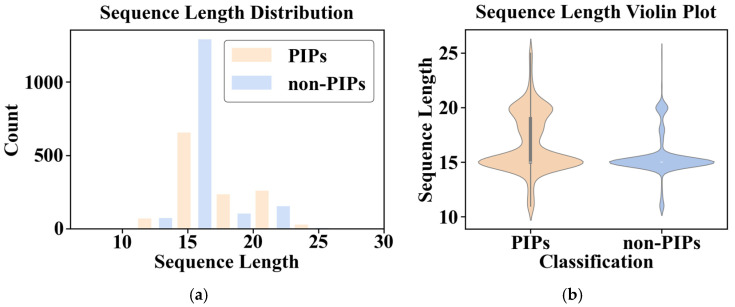
The statistical results of the data used in this work: (**a**) Sequence Length Distribution; (**b**) Sequence Length Violin Plot.

The statistical analysis reveals that PIP and non-PIP sequences exhibit similar length distributions (primarily 10–22 aa; median of 15 aa) and overall amino acid composition patterns, with minor but notable variations, as shown in Figure 4. Specifically, PIP sequences exhibit enrichment in leucine (L), arginine (R) and serine (S), while showing depletion in aspartic acid (D), glycine (G), proline (P) and threonine (T).

Comparative analysis reveals fundamental differences in the sequence architecture between PIPs and non-PIPs. We identified motifs ranging from 4 to 25 amino acids in both groups, as shown in Table 8. Further motif analysis indicates that their immunomodulatory functions are closely associated with specific physicochemical motifs, such as amino acid composition, charge distribution, and amphipathicity, reflecting distinct structural mechanisms and functional orientations between the two classes. This clear differentiation provides a theoretical foundation for the rational design of peptide-based drugs with precise immunomodulatory activities.

In PIPs, two characteristic motifs are identified, both displaying strong targeting propensity and interaction tendencies. Pro-inflammatory Motif 1 is enriched with glutamine (Q) clusters. The side chain amide groups of glutamine serve as excellent hydrogen bond donors and acceptors, forming a polar, neutral, flexible, and hydrogen bond-rich “interaction platform”, which likely facilitates specific recognition and binding to pro-inflammatory receptors. For instance, hydrophilic interactions dominate the interaction on the buried surfaces of IL-18/IL-18Rα complex, a pattern that is broadly conserved among IL-1 family members [14]. The presence of phenylalanine (F) and leucine (L) introduces hydrophobic patches, potentially facilitating initial peptide anchoring at the binding interface. Pro-inflammatory Motif 2 is rich in hydrophobic and aromatic amino acids. Its dense hydrophobic and aromatic side chains constitute an interaction hotspot that may guide binding to cytokines or receptors. The central tryptophan (W) anchors into the receptor pocket via hydrophobic and cation–π interactions, while tyrosine (Y) contributes to hydrogen-bonding networks, key forces in protein interactions. Although proline (P) is not generally enriched across PIPs, its strategic placement can introduce turns within the framework, reorienting adjacent residues, which is critical for shaping specific tertiary structures and binding interfaces. Moreover, variations at certain positions are tolerated or even favored, enabling fine-tuning of affinity and specificity toward different pro-inflammatory receptors across varying physiological environments.

In contrast, the motifs identified in non-PIPs follow entirely different structural principles. Non-pro-inflammatory Motif 3 features a glycine (G) and proline (P) backbone, with glycine recurring regularly and specific positions accommodating other amino acids such as glutamic acid (E) and alanine (A). This motif has previously been reported as a typical anti-inflammatory peptide motif [15]. In non-pro-inflammatory Motif 4, positively charged arginine (R), lysine (K), and histidine (H) are positioned on one side, and negatively charged glutamic acid (E) is located centrally. These charged residues potentially influence interactions through electrostatic repulsion or competitive binding.

DDE encoding incorporates the natural occurrence probabilities of amino acids derived from the genetic codon table, offering a representation that accounts for codon usage bias—unlike simple frequency-based methods such as AAC or dipeptide composition. By quantifying the statistical significance of deviations between observed and expected dipeptide frequencies, DDE captures evolutionary and translational constraints inherent in peptide sequences. In contrast to standard dipeptide composition methods like GDPC, which merely report empirical frequencies, DDE provides a biologically informed, statistically robust, and computationally efficient representation of compositional properties.

Complementarily, ESM-2 captures high-level evolutionary and structural features through its deep transformer architecture, enabling the modeling of contextual and fold-specific information. The attention patterns in ESM-2 not only correspond to protein tertiary structure but also lead to a much deeper understanding of structural properties. Trained with masked language modeling (MLM), ESM-2 captures meaningful semantic relationships and long-term dependencies between amino acids.

The fusion of DDE and ESM-2 embeddings therefore achieves a synergistic integration of compositional attributes with semantic representations, enriching the feature space for enhanced discriminative capability. Building upon this hybrid representation, the global refinement strategy further strengthens feature integration by facilitating cross-modal alignment between DDE features and ESM-2 embeddings. This approach mitigates information loss associated with premature feature pruning while achieving a high compression ratio of 15.34 (reducing 2960 dimensions to 193), which directly enhances computational efficiency and minimizes memory footprint.

The generalization capability of models in practical applications necessitates rigorous evaluation using an independent test set. In the assessment on the independent test dataset, the overall performance of models generally declined compared to cross-validation, underscoring the critical generalization capability. The synergistic effect between feature refinement methods and algorithms critically determines model performance, and generalization capability must be stringently validated through independent testing. Models using Dual-channel features generally experienced performance degradation, indicating weaker generalization capability.

The comparative analysis between DDE and CKSAAP revealed important insights into feature encoding selection. While CKSAAP-based models demonstrated superior performance in cross-validation scenarios (achieving higher Sensitivity and AUC), DDE-based models exhibited significantly better generalization capability in independent testing. This divergence highlights that initial marginal differences in feature evaluation can indeed be amplified through the modeling pipeline, but the direction of amplification depends on the evaluation context. The superior stability of DDE across different datasets suggests it captures more robust and transferable patterns for PIP prediction. Our finding that subtle differences in feature encoding can lead to divergent model behaviors has important implications for computational biology methodology. The 0.01 sensitivity difference observed in the initial feature evaluation amplified to substantial performance variations in final models, underscoring the importance of evaluating features within the complete modeling pipeline rather than in isolation.

Importantly, the highest-dimensional features without refinement (ESM + DDE, 2960 dimensions) did not surpass other refinement methods across all algorithm combinations, indicating that feature dimensionality alone is not a decisive factor for performance. Particularly, the combination of ESM + DDE features with DT performed the poorest with Acc of 0.602 and MCC of 0.205, further substantiating the risk of overfitting associated with high-dimensional feature spaces. The benefits of Decision Trees include their interpretability and low requirements for data preprocessing. However, a major drawback is their propensity to overfitting, a limitation that motivated the development of ensemble methods to enhance robustness and accuracy.

Across all feature spaces, tree-based ensemble models (particularly Random Forest (RF), XGBoost, and CatBoost) significantly outperformed the single Decision Tree (DT) and AdaBoost algorithms, highlighting the effectiveness of ensemble learning. In RF, two strategies—bagging and random feature selection—enhance the model’s generalization abilities and robustness against overfitting. In contrast to bagging techniques which build trees independently, boosting is another ensemble approach which trains learners sequentially. Relative optimizations expand upon traditional gradient boosting, resulting in enhanced computational efficiency and scalability. Particularly, CatBoost is an advanced gradient boosting frameworks which trains learners sequentially and handles feature processing automatically, eliminating the necessity for manual preprocessing. Critically, the CatBoost algorithm employs symmetric (oblivious) tree structures to model complex feature interactions, and is further strengthened by gradient bias reduction mechanisms. Thus, it excels in handling high-dimensional data and demonstrates robustness to dataset shift. The capability for native categorical feature processing, combined with its sophisticated training algorithm, allows CatBoost to achieve superior predictive accuracy with minimal hyper parameter tuning.

The experimental results indicate that both CNN and Att-BiLSTM architectures were outperformed by the CatBoost classifier, despite their acknowledged representational power. Several interrelated factors may explain such outcome.

Deep Learning models, such as CNN and Att-BiLSTM, generally require large-scale datasets to fully learn underlying feature representations and avoid overfitting. In contrast, our benchmark dataset is of small-to-medium size, and CatBoost is particularly renowned for its high efficiency and robustness on data of this scale. Moreover, the feature space used in this work is inherently tabular, especially after feature selection and refinement. Gradient Boosting Decision Tree (GBDT) methods like CatBoost are explicitly well-suited for such data. CNNs, on the other hand, inherently excel with data possessing spatial or topological structure (e.g., images, sequences), and the Att_BiLSTM is primarily effective for capturing long-range temporal dependencies. This inherent inductive bias may make them suboptimal for generic tabular data where feature relationships are neither sequential nor spatial. The use of ESM-2 for feature extraction provides a further explanation. Given that ESM-2 is built on the Transformer architecture, its self-attention mechanisms are inherently designed to capture complex, long-range dependencies and global context in protein sequences. Consequently, applying additional deep neural networks (CNNs or Att-BiLSTMs) to these pre-processed representations may produce only marginal improvements, as the most salient contextual patterns have already been encoded. CatBoost circumvents this limitation through its tree-based inductive bias, which is fundamentally different from that of neural networks. This methodological complementarity allows CatBoost to utilize the high-quality embeddings more effectively for tabular prediction, achieving superior results without introducing redundant deep learning layers. Additionally, the performance of deep learning models is highly sensitive to hyperparameter configurations. Although we conducted a search over key parameters, the scope was necessarily limited. A more exhaustive hyperparameter optimization or the use of more advanced architectures might yield improved performance. Nevertheless, CatBoost achieved strong performance with minimal hyperparameter tuning, demonstrating its advantage as a readily deployable and effective solution for PIP prediction.

Cat-PIPpred delivers significant advantages in computational efficiency, predictive stability, and holistic performance, addressing critical requirements in contemporary proteomic research. Consequently, when benchmarked against existing methods, Cat-PIPpred demonstrates that incorporating structural insights from ESM-2 and leveraging advanced machine learning architectures yields significant gains in predictive performance. Deep_AMPpred and AMPpred_MFA are general-purpose predictors, and thus have considerable limitations in accurately detecting PIP, underscoring the need for specialized predictors such as Cat-PIPpred and MultiFeatVotPIP (reliant on handcrafted compositional features).

Cat-PIPpred exhibits enhanced efficacy relative to current PIP prediction methodologies due to two principal methodological and biological innovations.

Firstly, our framework employs a cross-modal feature fusion technique, significantly benefiting from the pre-trained ESM-2 features. In contrast, existing PIP predictors, such as MultiFeatVotPIP, predominantly depend on conventional compositional features. Although ProIn-Fuse integrates structural data, it is constrained to merely eight types of handcrafted structural descriptors, whereas our model assimilates high-dimensional evolutionary and structural representations derived from the ESM-2 model. ESM-2 represents a cutting-edge large-scale protein language model that effectively captures the fundamental principles governing protein evolution, structure, and function. The 2560-dimensional ESM-2 embedding utilized in this study provides a comprehensive, context-sensitive representation of each peptide. By amalgamating the ESM-2 embedding with traditional features, Cat-PIPpred formulates a more integrated and informative feature set, thereby enhancing the descriptor for PIP and facilitating more precise identifications. This further emphasizes the biological insight that pro-inflammatory functionality is intrinsically linked to the compositional, structural, and evolutionary attributes of peptide sequences.

Secondly, Cat-PIPpred utilizes the CatBoost classifier, which excels in managing categorical features and mitigating overfitting. The gradient boosting framework of CatBoost demonstrates superior generalization capabilities and robustness. In contrast, ProIn-Fuse combines eight single encoding-based random forest models linearly, while MultiFeatVotPIP employs a soft voting ensemble approach with five principal learners (AdaBoost, XGBoost, RF, GBDT, and LightGBM). The marginal enhancement of MultiFeatVotPIP compared to its individual components indicates a lack of diversity among its constituent models.

**Figure 5:**
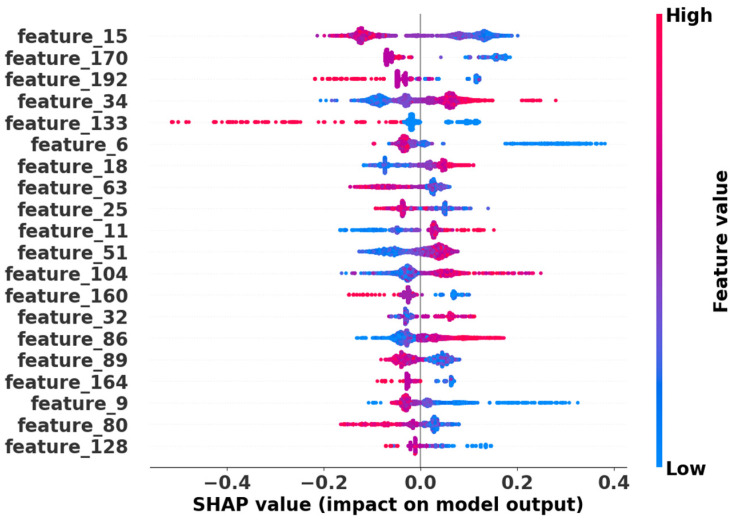
A SHAP summary plot of the top 20 features.

Figure 5 presents the SHAP (SHapley Additive exPlanations) summary plot of the top 20 features among the retained 193 features in Cat-PIPpred. The features are ordered by their mean absolute SHAP values, representing their overall importance in the model. This ranking reflects the average magnitude of feature contributions across all predictions in the dataset. The results demonstrate that these selected features significantly influence the model’s predictions, with higher absolute SHAP values indicating greater feature importance.

The distribution of points reveals the directionality of each feature’s impact. Specifically, the horizontal spread of points along the SHAP value axis indicates the magnitude and consistency of each feature’s impact across different samples, with wider distributions signifying stronger and more variable influence on predictions.

Our analysis identified distinct patterns of feature contributions: for features showing positive correlation (e.g., Feature 34, 11, and 86), red points (high feature values) cluster on the right (positive SHAP values) and blue points on the left, indicating that higher feature values increase interaction probability. Conversely, for features showing negative correlation (e.g., Feature 192, 133, and 160), red points concentrate on the left while blue points are distributed on the right, suggesting that higher feature values decrease prediction scores.

The clear separation of positive and negative impacts across different features demonstrates the model’s ability to capture biologically relevant determinants for predicting PIPs. This interpretability analysis strengthens the biological plausibility of our predictions and provides mechanistic insights into the factors driving pro-inflammatory activities.

As evidenced, this framework establishes a new paradigm for specialized peptide function prediction by synergizing pre-trained language models with evolution-aware feature engineering. This architecture explicitly accounts for the computational complexity inherent in the refinement process while maintaining robust predictive performance.

## 4. Materials and Methods

### 4.1. Data Collection

The dataset utilized in this study is provided by Yan et al., publicly accessible from https://github.com/ChaoruiYan019/MultiFeatVotPIP (accessed on 20 February 2025) [9]. It consists of human and mouse peptides sourced from the IEDB database [16]. Nine clinically significant pro-inflammatory cytokines are specifically focused: IL-1α, IL-1β, IL-6, TNF-α, IL-12, IL-23, IL-8, IL-18, and IL-17. Peptides triggering any of these nine cytokines are labeled as positive samples, while other peptides are designated as negative samples. Peptide sequences outside the 5–25 amino acid length range were excluded. CD-HIT was applied to reduce redundancy with the threshold of 0.60. The final dataset comprised 2872 training samples (1245 positive, 1627 negative) and 342 test samples (171 positive and negative each). All cross validation experiments are based on the same training dataset, and all independent tests are based on the same test dataset. For comprehensive details regarding the data collection and processing procedures, please refer to the work of Yan et al. [9].

### 4.2. Feature Extraction and Refinement

Both pre-trained feature and composition, property-based feature are utilized in this work, so as to enrich feature space [17]. DDE, AAC, CKSAAP, GDPC, and CTDC encoding are evaluated, among which DDE is finally chosen. The extracted feature vectors are further processed to optimize model performance. For handling missing values, mean imputation is employed, replacing missing entries with the mean value of corresponding feature. This strategy effectively preserves the overall statistical structure of the dataset while introducing minimal bias. To address feature scale variations, all features are standardized to a zero-mean, unit-variance distribution. This standardization process ensures uniform feature contribution and enhances numerical stability.

#### 4.2.1. Pre-Trained Feature (ESM-2)

ESM-2 is a series of transformer-based protein language models capable of unsupervised learning evolutionary patterns from millions of protein sequences [10]. In ESM-2, an input amino acid sequence is first tokenized into an integer-indexed sequence. This sequence is then processed through an embedding layer, followed by multiple Transformer encoder layers with multi-head self-attention mechanisms and feed-forward neural networks. The last encoder layer outputs contextualized representations for each amino acid position, which can be pooled to generate a global contextual feature of the entire sequence. In this study, we use esm2_t33_650M_UR50D, a medium-sized ESM-2 variant with 33 layers and 650 million parameters. This model achieves an optimal balance between predictive performance and computational efficiency, generating 2560-dimensional feature vectors that are well-suited for downstream prediction tasks.

#### 4.2.2. Composition and Property-Based Feature

The compositional feature representation is chosen out of five sequence-based encoding methods, including DDE, AAC, GDPC, CTDC, and CKSAAP.

Dipeptide Deviation from Expected Mean (DDE) encoding quantifies the statistical deviation of observed dipeptide frequencies from their theoretical expectations in protein sequences. It transforms a protein sequence P into a 400-dimensional feature vector representing all possible ordered dipeptide pairs of the 20 natural amino acids:

$$\mathrm{DD}E_{P\;}=\;\{\mathrm{DD}E_{\left(1\right)},\ldots ,\ldots ,\mathrm{DD}E_{\left(400\right)}\}$$

In detail, the theoretical frequency of amino acid x is

$$f\left(x\right)=\frac{C_{x}}{C_{N}}\;,$$

with $C_{N}$ representing the number of possible codons (excluding the three stop codons), $C_{x}$ representing the numbers of codons corresponding to x. For dipeptide $i\;=\;(i_{1},i_{2})$, its theoretical mean $T_{m\left(i\right)}$, theoretical variance $T_{v\left(i\right)}$ and dipeptide composition $D_{c\left(i\right)}$ are calculated according to

$$T_{m\left(i\right)}\;=\;f\left(i_{1}\right)\times \;f\left(i_{2}\right),$$

$$T_{v\left(i\right)}\;=\;\frac{T_{M\left(i\right)}\left(1-T_{M\left(i\right)}\right)}{N},$$

$$D_{c\left(i\right)}\;=\;\frac{n_{i}}{N},$$

where $n_{i}$ is the occurrence count of dipeptide i in P, and $N\;=\sum_{i=1}^{400}n_{i}$ is the total number of dipeptides in P. The standardized residual of dipeptide i is

$$\mathrm{DD}E_{\left(i\right)}=\frac{D_{c\left(i\right)}-T_{m\left(i\right)}}{\sqrt{T_{v\left(i\right)}}}.$$

Amino Acid Composition (AAC) calculates the occurrence frequency of each standard amino acid in a protein sequence. It provides simple global composition information of 20 dimensions. K-Spaced Amino Acid Pairs (CKSAAP) counts all possible amino acid pairs separated by k residues (in this article, the value of k is set to 5). It captures local interaction patterns while maintaining some sequence order information. Grouped Di-Peptide Composition (GDPC) is a condensed version of the conventional dipeptide composition, which groups amino acids based on their physicochemical or structural properties, including aliphatic, aromatic, positively charged, negatively charged, and uncharged. The frequencies of dipeptides formed by these groups form a 25-dimensional feature vector. Composition, Transition, and Distribution of Codons (CTDC) captures the statistical patterns of codon usage in protein-coding sequences by integrating three components: the compositional feature is computed as the normalized frequency of each codon; the transition feature quantifies the dinucleotide dynamics between consecutive codons by measuring the probabilities of purine-pyrimidine (R-Y) transitions at the first nucleotide positions of adjacent codons; the distributional feature characterizes the positional trends of codons by evaluating their differential enrichment across three sequence segments: the initial 25% (5′-terminal region), middle 50% (central region), and final 25% (3′-terminal region).

#### 4.2.3. Feature Refinement

Three different feature dimensionality refinement strategies are designed in this work: two-stage refinement, global refinement, and dual-channel global refinement. (1) Two-stage refinement: ESM-2 pre-trained features undergo dimensionality reduction Via Lasso regression in the first stage. The optimized ESM-2 features were subsequently fused with the DDE features, yielding a final 526-dimensional vector. (2) Global refinement: ESM-2 feature and DDE feature were integrated at first, and LASSO regression was applied to the fused feature. After sparsity-constrained feature space compression, the final feature vector was 193-dimensional. (3) Dual-channel global refinement: LASSO regression was applied to the ESM-2 feature and DDE feature, respectively, and the regularization coefficient α was optimized independently for each channel. The dual-channel features were fused. Then, LASSO constraint was implemented to refine a final feature vector, which was 210-dimensional. Among three strategies, such dual-channel global refinement is the most complex.

### 4.3. Classification Algorithm

In the realm of machine learning, various classification algorithms play a crucial role in data analysis and predictive modeling. In this work, we try a traditional machine learning algorithm (DT) and several ensemble learning algorithms. Specifically, a bagging approach (RF) and three boosting methods (AdaBoost, CatBoost, and XGBoost) are evaluated. Therein, RF, XGBoost, and CatBoost are tree-based ensemble models.

Decision Trees (DT) serves as a supervised learning technique applicable to both classification and regression tasks [18]. This algorithm operates by recursively dividing the dataset into smaller subsets based on feature values, ultimately creating a hierarchical tree structure where internal nodes signify decision rules, branches represent possible outcomes, and leaf nodes provide final predictions.

Random Forest (RF) emerges as a powerful and widely adopted ensemble learning algorithm that improves predictive performance by constructing multiple decision trees and combining their results [19]. Each decision tree is trained on a randomly selected subset of the training data Via bootstrap aggregation (bagging), which fosters diversity among the trees. Additionally, at each split, only a randomly chosen subset of features is considered, reducing the correlation between trees.

AdaBoost (Adaptive Boosting) is a representative boosting algorithm, which integrates multiple weak classifiers into a robust ensemble classifier [20]. It employs an iterative reweighting strategy that progressively increases the weights of misclassified samples, compelling subsequent learners to concentrate on challenging cases, while adaptively adjusting the weights of each weak learner based on its accuracy.

CatBoost (Categorical Boosting) trains learners sequentially and handles feature processing automatically. It utilizes oblivious (symmetric) decision trees and permutation-driven ordered boosting to ensure computational efficiency and model robustness. CatBoost is an advanced gradient boosting frameworks specifically designed to efficiently process heterogeneous datasets that include both numerical and categorical features [21].

XGBoost is an efficient end-to-end gradient boosting decision tree framework that introduces systematic optimizations to tackle significant computational and statistical challenges in large-scale machine learning [22]. It employs a sparsity-aware algorithm to manage sparse data and utilizes a theoretically justified weighted quantile sketch for approximate learning.

### 4.4. Performance Indicators

In this work, five common metrics are measured to evaluate the predictive model comprehensively, including sensitivity (Sn), specificity (Sp), accuracy (Acc), Matthew’s correlation coefficient (MCC) and the area under the receiver operating characteristic curve (AUC). The formulas are listed below:

$$\left\{\begin{array}{c} Sp\;=\;\frac{\mathrm{TN}}{\mathrm{TN}+\mathrm{FP}},\\ Sn\;=\;\frac{\mathrm{TP}}{\mathrm{FN}+\mathrm{TP}},\\ Acc\;=\frac{\mathrm{TP}+\mathrm{TN}}{\mathrm{TP}+\mathrm{TN}+\mathrm{FN}+\mathrm{FP}},\\ MCC\;=\frac{\mathrm{TP}\times \mathrm{TN}-\mathrm{FP}+\mathrm{FN}}{\sqrt{\left(\mathrm{FN}+\mathrm{TP}\right)\left(\mathrm{TN}+\mathrm{FP}\right)\left(\mathrm{TP}+\mathrm{FP}\right)\left(\mathrm{FN}+\mathrm{TN}\right)}}, \end{array}\right.$$

where TP, TN, FP and FN represent the number of true positives, true negatives, false positives and false negatives, respectively.

## 5. Conclusions

In this study, we developed Cat-PIPpred to address the critical need for accurate pro-inflammatory peptide (PIP) identification by synergistically integrating evolutionary-informed feature representations with deep structural embeddings. By leveraging the complementary strengths of DDE encoding and ESM-2 protein language modeling, this framework overcomes limitations of prior methods in capturing both compositional biases and high-level contextual features, thereby establishing a robust foundation for enhanced predictive performance and biological interpretability.

## Figures and Tables

**Table 1:** DDE-based comparison results of different feature refinements and algorithms using the five-fold cross validation test.

Feature Refinement	Dimension	Algorithm	Acc	Sn	Sp	AUC	MCC
ESM + DDE	2960	DT	0.653	0.521	0.754	0.667	0.283
		RF	0.687	0.508	0.824	0.730	0.354
		AdaBoost	0.657	0.521	0.762	0.682	0.292
		CatBoost	0.683	0.519	0.809	0.719	0.345
		XGBoost	0.684	0.529	0.803	0.730	0.347
Two-stage	526	DT	0.665	0.494	0.796	0.684	0.306
		RF	0.693	0.504	**0.837**	**0.745**	0.365
		AdaBoost	0.678	0.551	0.774	0.713	0.335
		CatBoost	0.688	0.523	0.814	0.728	0.354
		XGBoost	0.696	0.548	0.809	0.741	0.372
Global	193	DT	0.665	0.458	0.824	0.679	0.306
		RF	0.695	0.516	0.832	0.743	0.369
		AdaBoost	0.686	**0.568**	0.776	0.716	0.352
		CatBoost	0.691	0.52	0.821	0.738	0.361
		XGBoost	0.692	0.534	0.813	0.736	0.364
Dual-channel	210	DT	0.656	0.472	0.796	0.656	0.285
		RF	0.693	0.513	0.830	0.743	0.366
		AdaBoost	0.673	0.545	0.770	0.713	0.325
		CatBoost	**0.700**	0.540	0.822	0.741	**0.381**
		XGBoost	0.696	0.544	0.813	0.742	0.373

**Table 2:** DDE-based comparison results of different feature refinements and algorithms on the independent test dataset.

Feature Refinement	Dimension	Algorithm	Acc	Sn	Sp	AUC	MCC
ESM + DDE	2960	DT	0.602	**0.579**	0.626	0.634	0.205
		RF	0.637	0.515	0.760	0.678	0.284
		AdaBoost	0.646	0.561	0.731	0.676	0.297
		CatBoost	0.643	0.515	0.772	0.682	0.297
		XGBoost	**0.652**	0.532	**0.772**	**0.687**	**0.313**
Two-stage	526	DT	0.637	0.497	0.778	0.663	0.286
		RF	0.646	0.503	**0.790**	0.692	0.305
		AdaBoost	0.632	0.544	0.719	0.686	0.267
		CatBoost	0.635	0.503	0.766	**0.694**	0.279
		XGBoost	**0.655**	**0.556**	0.754	0.691	**0.316**
Global	193	DT	0.617	0.556	0.678	0.611	0.236
		RF	0.632	0.497	0.766	0.696	0.273
		AdaBoost	0.626	0.550	0.702	0.682	0.254
		CatBoost	**0.684**	**0.556**	**0.813**	**0.705**	**0.381**
		XGBoost	0.652	0.526	0.778	0.689	0.314
Dual-channel	210	DT	0.626	0.497	0.754	0.637	0.260
		RF	0.635	0.503	0.766	0.687	0.279
		AdaBoost	0.626	**0.544**	0.708	0.672	0.255
		CatBoost	0.635	0.509	0.760	**0.690**	0.278
		XGBoost	**0.643**	0.515	**0.772**	0.689	**0.297**

**Table 3:** CKSAAP-based comparison results of different feature refinements and algorithms using the five-fold cross validation test.

Feature Refinement	Dimension	Algorithm	Acc	Sn	Sp	AUC	MCC
ESM + CKSAAP	2560 + 2400	DT	0.581	0.397	0.722	0.582	0.125
		RF	0.642	0.381	0.843	0.674	0.255
		AdaBoost	0.610	0.480	0.710	0.632	0.194
		CatBoost	0.637	0.439	0.788	0.665	0.244
		XGBoost	0.647	0.463	0.787	0.675	0.267
Two-stage	126 + 2400	DT	0.586	0.337	0.778	0.573	0.136
		RF	0.666	0.357	**0.902**	0.718	0.316
		AdaBoost	0.634	0.510	0.729	0.666	0.245
		CatBoost	0.656	0.461	0.805	0.689	0.285
		XGBoost	0.666	0.516	0.782	0.712	0.310
Global	193	DT	0.585	0.204	0.876	0.569	0.111
		RF	0.668	0.382	0.888	0.754	0.317
		AdaBoost	0.697	0.598	0.773	0.748	0.377
		CatBoost	0.675	0.465	0.836	0.731	0.327
		XGBoost	**0.707**	**0.606**	0.784	**0.778**	**0.397**
Dual-channel	126 + 217	DT	0.587	0.385	0.742	0.585	0.137
		RF	0.672	0.438	0.851	0.716	0.321
		AdaBoost	0.656	0.547	0.740	0.703	0.293
		CatBoost	0.665	0.478	0.808	0.708	0.305
		XGBoost	0.667	0.498	0.796	0.712	0.310

**Table 4:** CKSAAP-based comparison results of different feature refinements and algorithms on the independent test dataset.

Feature Refinement	Dimension	Algorithm	Acc	Sn	Sp	AUC	MCC
ESM + CKSAAP	2560 + 2400	DT	0.535	0.480	0.591	0.586	0.071
		RF	0.623	0.421	0.825	0.638	0.268
		AdaBoost	0.617	0.491	0.743	0.665	0.242
		CatBoost	0.614	0.456	0.772	0.637	0.240
		XGBoost	0.602	0.439	0.766	0.656	0.217
Two-stage	126 + 2400	DT	0.620	0.468	0.772	0.625	0.252
		RF	0.632	0.357	**0.906**	0.700	0.315
		AdaBoost	0.649	**0.573**	0.725	0.691	0.302
		CatBoost	0.620	0.462	0.778	0.678	0.253
		XGBoost	0.649	0.515	0.784	0.688	0.310
Global	193	DT	0.564	0.240	0.889	0.565	0.169
		RF	0.640	0.386	0.895	0.706	0.326
		AdaBoost	0.646	0.532	0.760	0.709	0.300
		CatBoost	0.635	0.450	0.819	0.705	0.289
		XGBoost	**0.678**	0.567	0.789	**0.730**	**0.366**
Dual-channel	126 + 217	DT	0.541	0.550	0.532	0.541	0.280
		RF	0.629	0.433	0.825	0.669	0.291
		AdaBoost	0.643	0.556	0.731	0.700	0.238
		CatBoost	0.614	0.474	0.754	0.692	0.271
		XGBoost	0.632	0.509	0.754	0.684	0.082

**Table 5:** Comparison results of Cat-PIPpred and CKSAAP_XG-PIPpred.

Test	Methods	Acc	Sn	Sp	AUC	MCC
5-fold	Cat-PIPpred	0.691	0.520	**0.821**	0.738	0.361
	CKSAAP_XG-PIPpred	**0.707**	**0.606**	0.784	**0.778**	**0.397**
Independent test	Cat-PIPpred	**0.684**	0.556	**0.813**	0.705	**0.381**
	CKSAAP_XG-PIPpred	0.678	**0.567**	0.789	**0.730**	0.366

**Table 6:** The parameters and performances of deep learning architectures.

Architecture	Learn_Rate	Filters	Kernel_Size	Acc	Sn	Sp	AUC	MCC
CNN	0.0005	128	3	0.667	0.550	0.784	0.693	0.343
	0.0005	64	2	0.667	0.556	0.778	0.687	0.342
	0.001	64	2	0.681	0.556	0.807	0.703	**0.375**
Att_BiLSTM	-	-	-	0.565	**0.674**	0.456	0.608	0.135
Cat-PIPpred	-	-	-	**0.691**	0.520	**0.821**	**0.738**	0.361

**Table 7:** Comparision performance of Cat-PIPpred with other methods.

Test	Methods	Acc	Sn	Sp	AUC	MCC
5-fold	MultifeatVotPIP	**0.691**	0.508	**0.830**	0.718	0.360
	Cat-PIPpred	**0.691**	0.520	0.821	**0.738**	**0.361**
	AMPpred_MFA_mixed	0.549	0.587	0.513	0.567	0.1
	Deep_AMPpred	0.603	**0.629**	0.582	0.659	0.210
Independent test	MultifeatVotPIP	0.655	0.521	0.790	0.686	0.322
	Cat-PIPpred	**0.684**	**0.556**	0.813	**0.705**	**0.381**
	AMPpred_MFA	0.602	0.263	**0.942**	0.669	0.279
	Deep_AMPpred	0.615	0.514	0.693	0.640	0.210

**Table 8:** Motifs discovered by MEME [13].

Label		Motif	E-Value	Sites
PIP	1	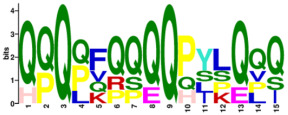	1.4 × 10^−7^	5
	2	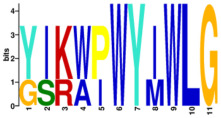	2.9 × 10^−4^	3
Non-PIP	3	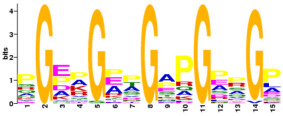	9.6 × 10^−89^	30
	4	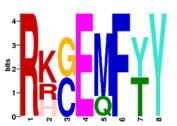	6.7 × 10^−4^	4

## Data Availability

The original data presented in the study are openly available in GitHub at https://github.com/ZhengJia0102/Cat-PIPpred (accessed on 27 October 2025).
